# Integrating incretin-based therapies into comprehensive obesity care: an EASO position statement

**DOI:** 10.1016/j.lanepe.2026.101840

**Published:** 2026-09-03

**Authors:** Jorge César Correia, Jennifer Lyn Baker, Emma Boyland, Luca Busetto, Lubomíra Fábryová, Tryggvi Helgason, Paolo Sbraccia, Euan Woodward, Volkan Yumuk, Zoltan Pataky, European Association for the Study of Obesity (EASO)

**Affiliations:** a Unit of Therapeutic Patient Education, WHO and EASO Collaborating Centre, Department of Primary Care Medicine, Geneva University Hospitals, Geneva, Switzerland; b Faculty Diabetes Centre, Faculty of Medicine, University of Geneva, Geneva, Switzerland; c European Association for the Study of Obesity, Teddington, UK; d Center for Clinical Research and Prevention, Copenhagen University Hospital-Bispebjerg and Frederiksberg, Frederiksberg, Denmark; e Department of Psychology, University of Liverpool, Liverpool, UK; f Department of Medicine, University of Padova, Padova, Italy; g Clinical and Physiological Nutrition, Slovak Medical University, Department of Diabetes and Metabolic Diseases, MetabolKLINIK, Lipid Clinic, MED PED Centre, Bratislava, Slovakia; h Children's Hospital, Landspítali University Hospital, Reykjavík, Iceland; i Dipartimento di Medicina dei Sistemi, Università di Roma “Tor Vergata”, Via Montpellier, 1, Rome, 00133, Italy; j Unit of Internal Medicine, Obesity Center, Policlinico Tor Vergata, Rome, Italy; k Division of Endocrinology, Metabolism and Diabetes, Department of Medicine Istanbul University-Cerrahpasa, Cerrahpasa Medical Faculty, Istanbul, Turkey

**Keywords:** Obesity, Incretin-based therapies, GLP-1 receptor agonists, Multidisciplinary care, Chronic disease management

## Abstract

Incretin-based therapies have transformed obesity treatment, producing substantial weight loss and benefits across cardiometabolic outcomes. However, translating therapeutic efficacy into sustainable population-level benefit remains challenging across European healthcare systems that vary in workforce capability, multidisciplinary care, reimbursement, access, and monitoring infrastructure. We describe this mismatch as the EASO Integration Paradox: therapeutic innovation has advanced more rapidly than the health-system structures required for its optimal, equitable, and sustainable implementation. In this EASO Position Statement, we propose the EASO Integration Framework, a model for integrating incretin-based therapies into comprehensive obesity care. The framework is organized around five interdependent pillars: Right Patient, Right Care, Right Workforce, Right Data, and Right Access. We also outline a European research and implementation agenda focused on real-world evidence, harmonised monitoring, workforce development, pharmacovigilance, and equitable access. The challenge is no longer whether incretin-based therapies should be used, but how they should be implemented responsibly within comprehensive obesity care pathways.

## Introduction

Obesity is one of the most pressing public health challenges facing Europe. Its prevalence has more than doubled among adults since 1990, and nearly 60% of adults in the WHO European Region are currently living with overweight or obesity.^1^ Similar trends have been observed among children and adolescents, highlighting the importance of adopting a life-course approach to obesity prevention and management.^1^ Obesity is increasingly recognized as a chronic, relapsing, adiposity-based disease associated with a broad spectrum of complications, including type 2 diabetes, cardiovascular disease, chronic kidney disease, metabolic dysfunction-associated steatotic liver disease (MASLD), several cancers, and impaired quality of life.^2^, ^3^, ^4^, ^5^

Incretin-based therapies have transformed the therapeutic landscape of obesity care. Semaglutide and tirzepatide achieve substantial weight reduction while improving cardiometabolic risk factors and obesity-related complications.^6^, ^7^, ^8^, ^9^, ^10^ Evidence of cardiovascular, renal, and hepatic benefits has further expanded their relevance across medical specialties.^9^^,^^11^^,^^12^

The recent WHO guideline on GLP-1-based therapies and updated EASO Framework for the Pharmacological Treatment of Obesity and its Complications reflect this shift while emphasizing that pharmacotherapy should be embedded within comprehensive obesity care.^2^^,^^13^

Rapid therapeutic progress, however, is creating new implementation challenges. Patient selection, multidisciplinary care, workforce preparedness, long-term monitoring, pharmacovigilance, affordability, and equitable access are increasingly important as treatment eligibility and prescribing expand. These challenges are particularly relevant in Europe, where obesity care infrastructure, reimbursement, specialist capacity, and access to multidisciplinary services vary substantially. Building on EASO's longstanding recognition of obesity as a chronic disease requiring multidisciplinary and person-centered care,^3^, ^4^, ^5^ we propose the EASO Integration Framework, a practical model for the responsible integration of incretin-based therapies into comprehensive obesity care across diverse European healthcare systems. The framework seeks to address what we describe as A European Research and Implementation Agenda: therapeutic innovation has advanced more rapidly than the health-system structures required to support its optimal, equitable, and sustainable implementation.

## The EASO Integration Paradox

Europe is experiencing a paradox in obesity care. Incretin-based therapies have advanced rapidly, with substantial effects on weight and expanding evidence of benefit across cardiovascular, renal, and hepatic disease. Yet the health-system structures required to support their optimal implementation have evolved more slowly. Variability in multidisciplinary obesity services, workforce capacity, reimbursement frameworks, long-term monitoring systems, and access pathways threatens to limit the translation of therapeutic efficacy into sustainable population-level benefit.

This implementation challenge is particularly important because obesity is a chronic disease that requires long-term management. Continued pharmacotherapy is associated with maintenance of weight loss and cardiometabolic improvements, whereas treatment discontinuation is frequently followed by weight regain.^14^, ^15^, ^16^, ^17^, ^18^ These observations reinforce the need to frame incretin-based therapies within a chronic disease model rather than as short-term interventions.

At the same time, cardiovascular, renal and hepatic outcome data are broadening involvement in obesity treatment beyond obesity medicine and endocrinology to cardiology, nephrology, hepatology, primary care, and other specialties.^9^^,^^11^^,^^12^ This expansion creates opportunities for earlier intervention and wider access but also increases the need for coordinated care and obesity-specific expertise. This is occurring against persistent gaps in obesity education and training across healthcare professions.^19^, ^20^, ^21^

Implementation also requires systems capable of evaluating effectiveness, safety, treatment persistence, and patient-centered outcomes in routine practice. Randomized trials establish efficacy and safety in selected populations, but real-world evidence and coordinated pharmacovigilance will be essential as treatment expands across more heterogeneous populations and healthcare settings.^22^, ^23^, ^24^ Finally, access remains highly variable. Differences in reimbursement, eligibility criteria, affordability, waiting times, and availability of specialist services risk widening existing inequalities if implementation is not explicitly designed around equity.^25^, ^26^, ^27^, ^28^, ^29^

Collectively, these challenges constitute the EASO Integration Paradox: therapeutic innovation has transformed what is possible in obesity care, but health systems have not yet fully adapted to deliver these advances comprehensively, equitably, and sustainably. Addressing this paradox requires moving beyond therapeutic adoption towards accountable integration. The **EASO Integration Framework** provides a structure for doing so.

## The EASO Integration Framework

Building on existing EASO frameworks for obesity management and emerging international guidance, we propose the EASO Integration Framework, a practical model for the responsible integration of incretin-based therapies into comprehensive obesity care across diverse European healthcare systems.

**Fig. 1:**
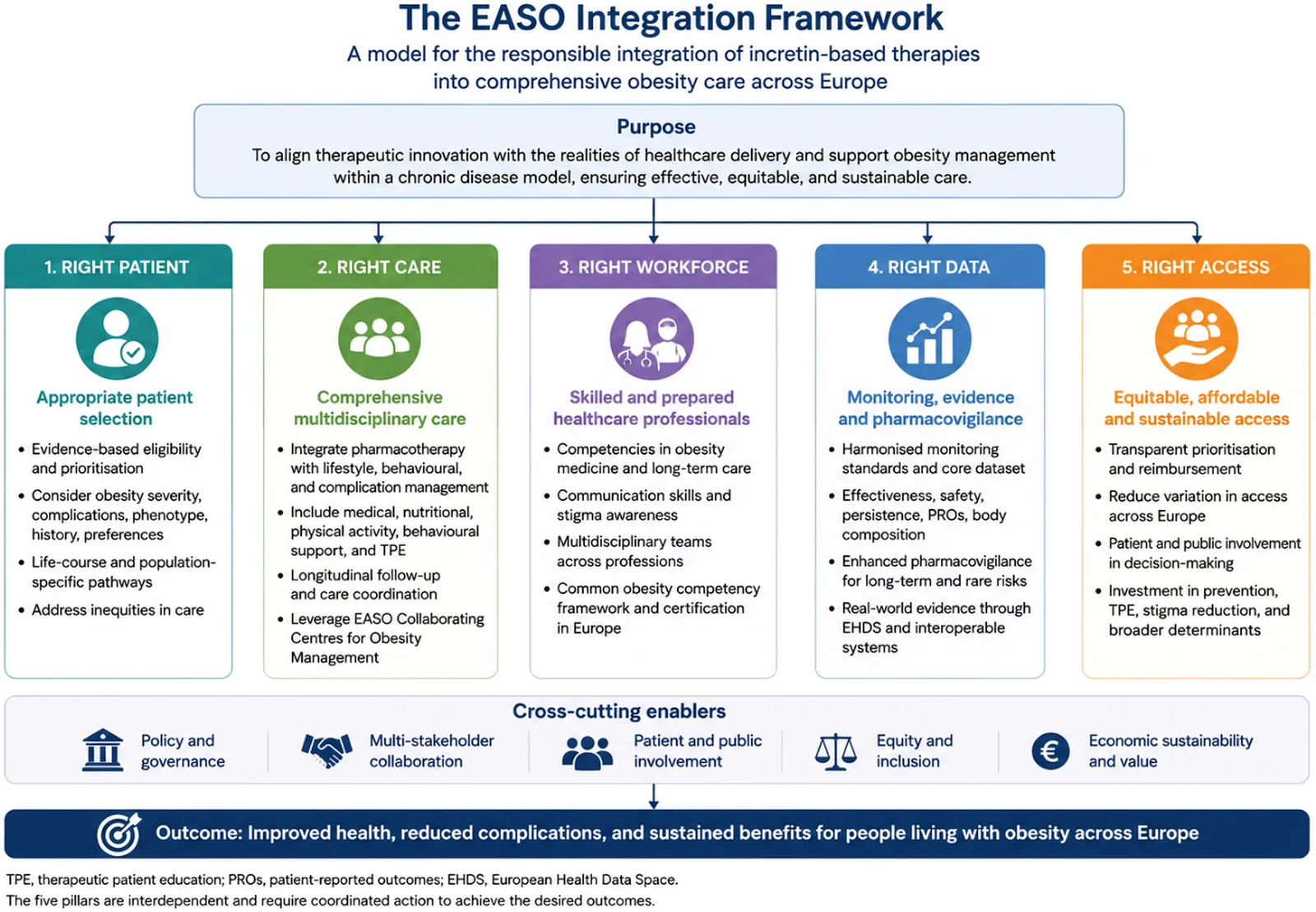
The EASO Integration Framework for incretin-based therapies. The EASO Integration Framework describes five interdependent pillars required for the responsible integration of incretin-based therapies into comprehensive obesity care across diverse European healthcare systems: Right Patient, Right Care, Right Workforce, Right Data, and Right Access. Together, these pillars aim to translate therapeutic efficacy into sustainable population-level benefit through improved health outcomes, safety, equity, and long-term sustainability. The framework is underpinned by a chronic disease model of obesity, person-centred and multidisciplinary care, a life-course approach, and continued investment in prevention and public health.

The framework is organized around five interdependent pillars: Right Patient, Right Care, Right Workforce, Right Data, and Right Access (Table 1; Fig. 1), providing a roadmap for translating therapeutic innovation into sustainable health outcomes while promoting equity.

**Table 1:** The EASO Integration Framework: domains, implementation instruments, and accountability metrics for the responsible integration of incretin-based therapies into obesity care.

Pillar	Key domains	Examples of implementation instruments	Examples of accountability metrics
Right patient	Appropriate patient selection and prioritisation	Phenotype-based treatment algorithms; assessment of obesity-related complications; shared decision-making; life-course approaches; individualised treatment goals	Proportion of eligible patients receiving treatment; representation of vulnerable populations; patient-reported experience measures; treatment persistence
Right care	Integration of pharmacotherapy within comprehensive obesity care	Multidisciplinary care pathways; therapeutic patient education (TPE); nutritional support; physical activity interventions; behavioural support; management of obesity-related complications; EASO Collaborating Centres for Obesity Management	Treatment adherence; quality-of-life measures; patient-reported outcomes; complication control; long-term maintenance of weight and health benefits
Right workforce	Workforce capability and multidisciplinary competencies	Harmonised competency frameworks; multidisciplinary training programmes; continuing professional development; ongoing EASO educational initiatives; emerging EASO Obesity Care Certification programme; multidisciplinary teams involving health and community professionals together with trained patient experts	Number of professionals receiving obesity-specific training; multidisciplinary team availability; competency assessments; access to specialist expertise
Right data	Monitoring, pharmacovigilance, and real-world evidence generation	Harmonised monitoring standards and core outcome sets; registries; pharmacovigilance systems; body composition assessment; patient-reported outcome measures; European Health Data Space interoperability	Treatment persistence; adverse event reporting; body composition outcomes; registry participation; quality indicators; real-world effectiveness measures
Right access	Equity, affordability, and sustainability	Reimbursement frameworks; prioritisation strategies; patient and public involvement; health-economic evaluation; shared-care models; policies aimed at reducing disparities between healthcare systems	Treatment uptake across socioeconomic groups; waiting times; geographical variation in access; cost-effectiveness measures; equity indicators

EHDS = European Health Data Space; EASO = European Association for the Study of Obesity.

### Pillar 1. Right patient

Appropriate patient selection requires transparent, evidence-based approaches to eligibility and prioritization. Consistent with the EASO pharmacological treatment framework, decisions should consider obesity severity and complications, clinical phenotype, treatment history, overall health, patient preferences, and anticipated benefit rather than BMI alone.^2^, ^3^, ^4^, ^5^ As access expands, healthcare systems must balance clinical need, expected benefit, and available resources while avoiding inequities in care.

Treatment should also be adapted across the life course, as goals, monitoring, and risk–benefit considerations differ in children and adolescents, reproductive life stages, and older adulthood. Age- and life-stage-specific pathways should therefore replace a uniform prescribing model, with particular attention to populations underrepresented in clinical trials and long-term outcome studies.

### Pillar 2. Right care

Incretin-based therapies should be integrated within comprehensive obesity care rather than delivered as stand-alone interventions. Given the biological, behavioral, psychological, social, and environmental determinants of obesity, care pathways should integrate pharmacotherapy with nutritional care, physical activity promotion, behavioral support, therapeutic patient education (TPE), and management of obesity-related complications.^30^

TPE is particularly important within this context. It can support realistic treatment expectations, shared decision-making, adverse-effect management, adherence, and long-term self-management. More broadly, multidisciplinary pathways can improve coordination and support outcomes beyond weight reduction, including health, function, and quality of life. Existing EASO Collaborating Centers for Obesity Management may provide valuable infrastructure for piloting, evaluating, and disseminating integrated models of care across diverse European healthcare systems. Such models may also serve as platforms for generating real-world evidence regarding the most effective approaches to delivering obesity care at scale.

### Pillar 3. Right workforce

Therapeutic innovation has outstripped formal training in obesity medicine, which remains limited across undergraduate, postgraduate, and continuing professional development programmes in many European countries.^19^, ^20^, ^21^^,^^31^ Responsible implementation requires competencies spanning obesity pathophysiology and assessment, pharmacotherapy, shared decision-making, behavioral support, therapeutic patient education, adverse-event management, long-term care, communication, and weight-stigma reduction.

Workforce development should extend beyond traditionally recognized medical professions and involve the broad range of health and community professionals who contribute to obesity prevention and management, together with trained patient experts, reflecting the multidisciplinary nature of effective obesity management.

Europe should work towards a common obesity competency framework applicable across healthcare professions. Building on ongoing EASO educational initiatives and the emerging Obesity Care Certification programme, such an approach could support harmonization of competencies in obesity prevention, diagnosis, pharmacological management, communication, stigma reduction, and long-term care across European healthcare systems.

### Pillar 4. Right data

Responsible implementation requires harmonized monitoring of effectiveness, safety, treatment persistence, and patient-centered outcomes beyond body weight alone.^22^

Core elements of monitoring should include body weight, measures of central adiposity such as waist circumference and waist-to-height ratio, cardiometabolic risk factors, treatment adherence, adverse events, discontinuation reasons, patient-reported outcomes, and, where feasible, body composition assessment.^32^^,^^33^ Future work should aim to establish harmonized core outcome sets for obesity pharmacotherapy, facilitating benchmarking and improving comparability across studies and healthcare systems.

Pharmacovigilance should address emerging priorities including gastrointestinal tolerability, gallbladder disease, body composition changes, bone health, perioperative management, pregnancy-related exposure, and mental health outcomes.^23^^,^^24^ Long-term registries and coordinated surveillance systems will play an increasingly important role in identifying rare adverse events and informing future clinical guidance.

Integration with emerging initiatives such as the European Health Data Space (EHDS) could support interoperability between registries, healthcare systems, and research networks, facilitating large-scale real-world evidence generation, benchmarking, and pharmacovigilance across Europe.^34^

### Pillar 5. Right access

Access to incretin-based therapies varies substantially across Europe because of differences in reimbursement policies, eligibility criteria, waiting times, access to specialist services, and affordability of treatment.^25^, ^26^, ^27^, ^28^, ^29^

Responsible implementation therefore requires transparent reimbursement frameworks, evidence-informed prioritization strategies, patient and public involvement, and ongoing health-economic evaluation to ensure that incretin-based therapies contribute to reducing rather than widening health inequalities.

People living with obesity should be actively involved in the development of prioritization frameworks, service design, and implementation strategies. Meaningful patient and public involvement can help ensure that access policies remain acceptable, responsive to patient needs, and aligned with real-world treatment experiences.

At the same time, the success of incretin-based therapies should not lead to a narrowing of obesity care around pharmacological treatment alone. Responsible implementation requires continued investment in prevention, health promotion, therapeutic patient education, stigma reduction, and action on the broader determinants of health. Pharmacotherapy should complement and be delivered alongside broader public health and healthcare-system strategies aimed at reducing the burden of obesity across Europe.

Together, these five pillars provide a practical framework for integrating incretin-based therapies into comprehensive obesity care. They recognize that long-term success depends not only on therapeutic efficacy but also on the systems, professionals, and policies that support implementation. By moving beyond therapeutic adoption towards accountable integration, European healthcare systems have an opportunity to maximize the benefits of these therapies while promoting equity, sustainability, and person-centered care.

## A European research and implementation agenda

Although the efficacy of incretin-based therapies is well established, important questions remain regarding their long-term population and health-system impact. Europe therefore needs a coordinated research and implementation agenda focused on real-world effectiveness, sustainability, equity, safety, and health-system performance.

### Generating real-world evidence for integrated obesity care

Although clinical trials have demonstrated substantial benefits of incretin-based therapies, less is known about their effectiveness when delivered through different models of care. Future research should prioritize pragmatic trials, registry-based studies, and implementation studies comparing pharmacotherapy-only approaches with integrated multidisciplinary pathways incorporating nutritional care, physical activity support, behavioral interventions, therapeutic patient education, and management of obesity-related complications.^22^^,^^30^

Outcomes should extend beyond weight loss and include body composition, cardiometabolic health, physical function, treatment persistence, quality of life, patient experience, healthcare utilization, and long-term maintenance of benefits. Understanding how different models of care influence these outcomes will be essential for informing future service design.

Existing EASO Collaborating Centers for Obesity Management may provide an ideal infrastructure for piloting and evaluating innovative care pathways across diverse European healthcare systems while facilitating cross-country comparisons and knowledge exchange.

### Developing harmonized monitoring standards and data infrastructure

The absence of harmonized monitoring standards limits the ability to evaluate implementation and compare outcomes across healthcare systems. A European minimum dataset and harmonized core outcome set for obesity pharmacotherapy should be developed to support benchmarking, quality improvement, pharmacovigilance, and real-world evidence generation.

Such a dataset could include anthropometric measures, measures of central adiposity, cardiometabolic risk factors, treatment adherence, adverse events, discontinuation reasons, patient-reported outcomes, and, where feasible, body composition measures. Harmonization would facilitate comparison between countries and healthcare settings while supporting more robust evaluation of treatment effectiveness at scale.

The European Health Data Space (EHDS) provides an important opportunity to improve interoperability between healthcare systems, registries, and research networks, supporting coordinated monitoring, implementation research, and post-marketing surveillance across Europe.^34^

### Strengthening pharmacovigilance and long-term safety evaluation

As incretin-based therapies are used across broader populations and over longer durations, continued pharmacovigilance will be essential. Although current evidence supports a favorable safety profile, important questions remain regarding long-term treatment exposure, body composition changes, bone health, perioperative management, pregnancy-related exposure, and rare adverse events.^23^^,^^24^

Future European registries should therefore incorporate structured safety monitoring capable of identifying emerging signals and informing future clinical guidance. Particular attention should be paid to populations currently underrepresented in clinical trials, including children and adolescents, older adults, individuals with multiple comorbidities, people with neurodevelopmental conditions, individuals from diverse ethnic backgrounds, and those receiving long-term therapy.

Monitoring systems should also evaluate outcomes that matter to patients, including physical function, quality of life, treatment burden, and maintenance of health improvements over time.

### Building workforce capacity through competency-based education

Major gaps in obesity-related education persist across healthcare professions.^19^, ^20^, ^21^^,^^31^

Implementation research should evaluate the effectiveness of competency-based training programmes, multidisciplinary care pathways, and shared-care models across different healthcare settings. Particular attention should be given to educational strategies that improve communication skills, reduce weight stigma, support shared decision-making, and strengthen confidence in long-term obesity care.

Building on ongoing EASO educational initiatives and the emerging Obesity Care Certification programme, Europe should work towards a common competency framework that can be adapted across healthcare professions and healthcare systems. Such an approach could help ensure that advances in pharmacotherapy are matched by corresponding advances in workforce capability.

### Ensuring equity, affordability, and sustainability

Future research should evaluate reimbursement models, prioritization frameworks, health-economic outcomes, and the impact of different access policies across European healthcare systems.^25^, ^26^, ^27^, ^28^, ^29^

Given the large number of individuals potentially eligible for treatment, budget impact analyses and cost-effectiveness evaluations will play an increasingly important role in policy decisions. Research should also explore how different implementation strategies affect health inequalities and whether particular groups face disproportionate barriers to accessing care.

Patients and people living with obesity should be actively involved in these discussions. Understanding patient priorities, treatment experiences, and perceived barriers will be essential for developing implementation strategies that are acceptable, effective, and sustainable.

Recent maintenance studies highlight the importance of developing individualized long-term treatment strategies and underscore the need for further research into maintenance pathways, dose optimization, dose tapering approaches, and non-pharmacological support.^18^

Collaboration across European healthcare systems, professional societies, patient organizations, and research networks will be essential to deliver this agenda.

Collectively, these priorities provide a foundation for a European implementation agenda that moves beyond therapeutic adoption towards accountable integration. Generating evidence across these domains will be essential to ensuring that incretin-based therapies fulfil their potential as part of comprehensive obesity care while strengthening, rather than fragmenting, obesity care across Europe.

## Conclusion

Incretin-based therapies have transformed obesity treatment, but therapeutic innovation alone will not guarantee improved population health or reduced inequalities. The long-term impact of these therapies will depend on the ability of healthcare systems to support their responsible, equitable, and sustainable implementation.

The EASO Integration Framework (Right Patient, Right Care, Right Workforce, Right Data, and Right Access) provides a roadmap for moving from therapeutic adoption towards accountable integration within chronic, person-centered, and comprehensive obesity care. Europe is uniquely positioned to lead this next phase. Existing EASO frameworks, educational initiatives, certification efforts, and networks of Collaborating Centers for Obesity Management provide important foundations upon which future implementation can be built. Emerging opportunities such as the European Health Data Space may further support coordinated monitoring, pharmacovigilance, and real-world evidence generation across healthcare systems.

The challenge facing Europe is no longer whether incretin-based therapies should be used, but how they should be implemented to strengthen rather than fragment obesity care. The future of obesity care will be determined not only by the medicines we develop, but by the systems we build to deliver them.

## Contributors

JCC conceptualized the manuscript, conducted the literature review, developed the EASO Integration Paradox and EASO Integration Framework, and wrote the original draft. ZP contributed to conceptualization, project coordination, supervision, and critical revision of the manuscript. JLB, EB, LB, LF, TH, PS, EW, and VY contributed to the evaluation of the evidence, provided intellectual input, critically reviewed the manuscript, and revised successive versions of the article. All authors approved the final version of the manuscript and agreed to its submission.

## Declaration of generative AI use

During the revision of this manuscript, the authors used ChatGPT (OpenAI) to assist with language editing in order to improve clarity, as well as figure generation. All scientific content, interpretation, recommendations, and final editorial decisions were developed, critically reviewed, and approved by the authors, who take full responsibility for the manuscript.

## Declaration of interests

JCC reports honoraria from Novo Nordisk and Eli Lilly, with payments made to his institution, and support from Novo Nordisk for travel and congress registration. JLB reports consulting fees and honoraria from Novo Nordisk, with payments made to her institution, and serves as President-Elect and Trustee of the European Association for the Study of Obesity (EASO), without remuneration. EB declares no financial competing interests and serves as an EASO Trustee and Chair of a WHO Technical Advisory Group. LB reports consulting relationships with Novo Nordisk, Eli Lilly, Amgen, Boehringer Ingelheim, Recordati, Pfizer, and Roche, and honoraria from Rhythm. LF reports honoraria from Eli Lilly, Novo Nordisk, Zentiva, and Boehringer Ingelheim; participation on advisory boards for Eli Lilly, Novo Nordisk, and Boehringer Ingelheim; and unpaid leadership roles as President of the Slovak Obesity Association, EASO Vice President, Chair of the Obesitology Section of the Slovak Diabetes Society, and member of the Slovak Atherosclerosis Society. TH reports travel support from EASO to attend professional and scientific meetings in connection with his role as an EASO Trustee and serves as an EASO Trustee. PS reports consulting fees from Boehringer Ingelheim, Chiesi, Eli Lilly, Novo Nordisk, Roche, and Theras, and honoraria from Chiesi, Eli Lilly, Novo Nordisk, and Theras; all payments were made directly to him. EW declares no competing interests. VY serves as President and Trustee of EASO. ZP declares no competing interests.
